# Use of Physiologic Reasoning to Diagnose and Manage Shock States

**DOI:** 10.1155/2011/105348

**Published:** 2011-08-09

**Authors:** Geoffrey Lighthall

**Affiliations:** Department of Anesthesia, Stanford University School of Medicine, 300 Pasteur Drive H3580, Stanford, CA 94305, USA

## Abstract

Shock states are defined by stereotypic changes in well-known physiologic parameters. While these well-known changes provide a convenient entry point into further evaluation of patients in shock or at risk for shock, use of such physiologic evaluation is not commonly seen in clinical medicine. A formal description of physiologic reasoning in the diagnosis of shock states is presented in this paper. Included with this conceptual framework is a discussion of key tests or findings that can be used to differentiate between possible diagnoses, and the pairing of treatment strategies to distinct classes of physiologic abnormalities. It is hoped that the methodology presented here will demonstrate the primacy of physiologic reasoning in the diagnosis and treatment of hemodynamic instability. Advantages of this method are speed and accuracy, efficient use of resources, and mitigation against sources of medical errors.

## 1. Introduction

Despite what appears to be rather universal teaching of principles of cardiovascular physiology in the didactic phase of undergraduate medical education, the application of such knowledge in patient care is often sparse. A classic example is the diagnosis and management of hemodynamic instability. The diverse set of clinical conditions that can be termed “shock states” are characterized by stereotypic perturbations in well-known physiologic relationships while resuscitation principles are based upon understanding and correcting their underlying parameters. Despite the simplicity offered by consideration of the underlying physiology of shock, it is rare to see trainees frame the management of hemodynamic instability in these terms.

Often, patient assessment appears to be governed more by a hit and miss approach where clinical context and suspicions lead to a series of diagnostic studies aimed to either rule in or out a specific hypothesis, or to narrow the range of possibilities. As a practical issue, this approach often delays therapy due to the reluctance to commence treatment prior to arriving at a diagnosis. Further, the approach may unnecessarily expose patients contrast dyes, X-rays, and other invasive procedures. The stabilization of hemodynamic compromise cannot tolerate a long series of investigations prior to institution of therapy, and, in fact, poorer patient outcomes have been associated with this approach [1–4]. In this paper, an approach to patient evaluation and stabilization founded purely on the principles of cardiovascular physiology is presented. It will be shown how the physiologic method can be used to (1) assure completeness of patient evaluation, (2) more efficiently narrow the list of potential diagnoses, (3) help prioritize the acquisition of diagnostic studies, and (4) better guide the choice of empiric therapy for shock states. 

Below, a general formulation of shock-related organ dysfunction will be presented, followed by a discussion of diagnostic evaluation and therapeutic planning. With this discussion, the reader will be provided with a framework for understanding the etiology and therapy of critical illness that is suitable for everyday use on the wards and in the ICU.

## 2. Pathophysiology of Shock States

For purposes of discussion, shock will be defined as a state of circulatory collapse of a magnitude that would lead to multiple organ dysfunctions if not rapidly corrected. While the basic classification of shock states into categories of cardiogenic, hypovolemic, and distributive shock is well known to all clinicians, these patterns are not always apparent to those caring for a patient. Rather, it is common to receive a call from a nurse or trainee reporting a patient with abnormal findings such as fever, tachycardia, or a drop in blood pressure. Starting from nonspecific findings such as these, a crucial part of patient evaluation includes determining whether the patient's findings are consistent with one of the shock states, and to have a reliable approach to this question that is consistent with the known pathophysiology. Accordingly, the risk for organ dysfunction arising from hemodynamic compromise can be traced to departure from the normal ranges in either one or both of the following physiologic relationships.

1. The autoregulatory curve describing the relationship between organ blood flow and mean arterial pressure [5].
2. The relationship between the rates of oxygen uptake (demand), and oxygen delivery (supply) [6–8].

**Figure 1:**
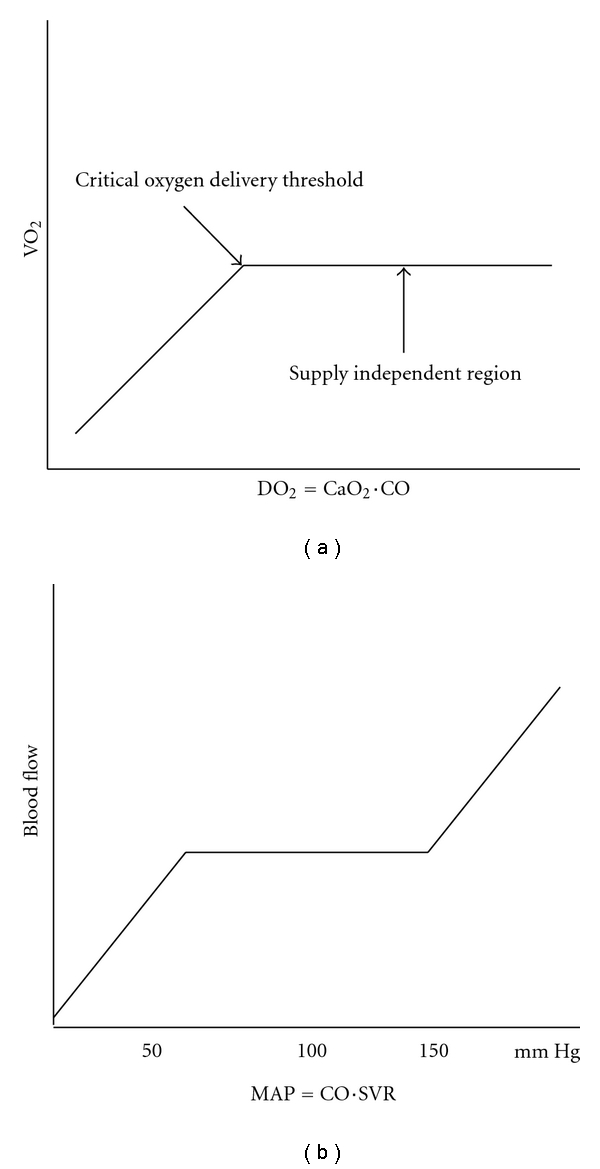
The key determinants of organ perfusion are depicted. In (a), the relationship between oxygen consumption (VO_2_) and delivery (DO_2_) is indicated. Patients usually function on the rightward side of the curve where an excess of oxygen is supplied relative to demand. As delivery decreases relative to consumption, the patient moves left on the curve. A decrease in central venous oxygen saturation accompanies leftward movement on the curve. In severe cases where delivery is unable to meet metabolic demands, the patient slips beneath the critical oxygen delivery threshold. Organ dysfunction and lactic acidosis are regarded as evidence of pathologic oxygen supply [6]. In (b), the autoregulatory curve (describing constancy of organ blood flow over a broad range or pressures) is shown. Some patients with chronic hypertension have curves shifted to the right relative to the normotensive curve shown here [5]. For both relationships shown, the flat horizontal portions indicate safe ranges, indicative of adequate organ blood flow and intact homeostatic mechanisms. Movement to the down sloping portions on the left indicates decompensation, placing the patient at risk for organ failure. *Abbreviations:* VO_2_, oxygen uptake/per minute; CaO_2_, oxygen content of arterial blood (mainly hemoglobin); CO, cardiac output; MAP, mean arterial pressure; SVR, systemic vascular resistance.

The graphic representations of these functions are presented in Figure 1, and their relevance to the familiar categorization of shock states is presented in Table 1. A drop in blood pressure below the auto regulatory threshold (MAP of 50) is insufficient for the kidneys, brain, and so forth to maintain flow to metabolically active regions. As shown by the MAP and D_O2_ equations in Figure 1, decreases in cardiac output will have quantitative and qualitative impacts on these variables. Even with a normal blood pressure, a low CO can cause significant impairment of oxygen delivery, at times so low that it is insufficient to meet current levels of oxygen consumption (oxygen debt).

The linkage between circulatory insufficiency and organ dysfunction involves the triggering of a state of malignant inflammation and microcirculatory dysfunction [9, 10]. Low oxygen tensions that may result from low delivery states such as hemorrhagic and cardiogenic shock can alter the production and elimination of reactive oxygen species as well as trigger the production of proinflammatory cytokines, cell adhesion molecules, nitric oxide, prostaglandins, and coagulation proteins via the NFkB pathway [11, 12]. A similar cascade of events is triggered by the innate immune system in response to microbial pathogens [13]. The proinflammatory/procoagulation phenotype phenotype is a final common pathway of all shock states, and the link between circulatory insufficiency and cellular effects ranging from reversible dysfunction, apoptosis, and death [9, 14, 15].

### 2.1. Using Physiologic Reasoning to Evaluate Shock

If the organ dysfunction resulting from most shock states can be traced to alterations in one or two key homeostatic relationships, then it seems reasonable that evaluation and diagnosis should involve a direct consideration of the same. Thus, for any patient exhibiting distress, change in solid organ function, or any suggestion of hemodynamic perturbation, the clinician bears the responsibility to consider the status of both the blood pressure/autoregulation and oxygen supply/demand relationships. Indeed, failure to evaluate these two relationships is a common source of misdiagnosis and poor patient outcomes in patients transferred from the ward to the intensive care unit [1, 16, 17].

**Figure 2:**
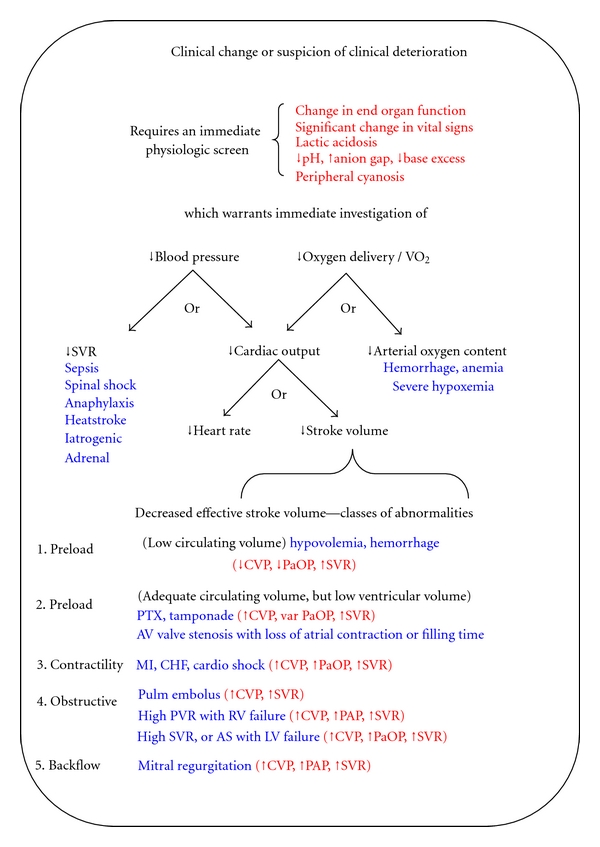
Dendrogram describing the constituents of mean arterial pressure and oxygen delivery in the context of suspected decompensation. For each key abnormality, physiologic variables are indicated in *black*, along with the main corresponding medical diagnoses indicated in *blue*. For each, key differentiating findings of laboratory or physiologic monitor data are presented in *red*. Abbreviations: VO_2_, oxygen uptake; DO_2_, oxygen delivery; CO, cardiac output; MAP, mean arterial pressure; SVR, systemic vascular resistance; CVP, central venous pressure; PaOP, pulmonary artery occlusion (wedge) pressure; PAP, pulmonary artery pressure; PTX, pneumothorax.

In practical terms, evaluation of the relationships noted above focuses on blood pressure and oxygen delivery. If an abnormality in the either maintenance of blood pressure or oxygen delivery is present, then further analysis is guided by the derivation of these terms according to the diagram in Figure 2. In the figure, physiologic parameters are highlighted in *black*, with key exam, monitor, or laboratory findings differentiating the categories noted in *red,* and possible diagnoses written in *blue*. 

To provide an example, assume you are called to evaluate a patient with a new onset of agitated delirium a few days after an abdominal operation. The patient is picking at his drains and indwelling devices; the pulse rate of 120 beats per minute is up from previous readings in the 80 s. The vital signs are BP: 121/85, temp: 36.5, SaO_2_: 95% on room air, and a respiratory rate of 26 per minute. The temptation of many would be to medicate the agitation with an antipsychotic and perhaps a benzodiazepine if alcohol withdrawal is suspected. Instead, the approach advocated here is to consider the pulse of 120 and a failing organ (cognitive dysfunction) as potential early warning signs of cardiorespiratory collapse. The scheme advocated here is to consider the physiology of oxygen delivery and blood flow prior to jumping to diagnostic conclusions, or at least to do so in parallel as a means of confirmation.

### 2.2. Evaluating Adequacy of Blood Pressure

Systolic blood pressure less than 90 mm Hg and mean pressures less than 60 mm are commonly used to indicate absolute hypotension. This is based less on rigorous testing and evidence than on tradition and classic large animal studies on autoregulation of blood flow. A baseline hypertensive patient would operate on a right-shifted antiregulatory curve, and may not have normal organ perfusion at mean pressures of less than 65–70 mm Hg. Retrospective analyses of trauma registries have supported the existence of age-related relative hypotension [18], and poorer outcomes at values previously considered normal [19]. Based on an aggregate of experience with septic shock, Marik and Lipman propose that patients should be considered to be hypotensive if after receiving 30 mL per Kg crystalloid infusion, decreases in systolic pressure are greater than 40 mmHg or drops of mean pressure are greater than 30 mmHg in normotensive patients [20]. Therefore, determination of adequacy of blood pressure often depends upon understanding a patient's usual range of pressures and the magnitude of acute change. Review of vital signs obtained in the outpatient setting or preoperative visit are quite helpful in this regard. Patients without clinic notes and charts may be more difficult to evaluate, but the presence of renal disease, left ventricular hypertrophy, and patient's own histories can provide some clues. Pressures noted on admission or obtained in the emergency department are *not* likely to reflect a patient's true baseline.

### 2.3. Evaluating Oxygen Supply/Consumption

Continuing with the case above, if the patient does not seem to be way off his baseline blood pressures, does this mean the patient's risk assessment is complete? No, an evaluation of oxygen supply demand status is necessary. Acute drops in arterial blood pH, changes in anion gap, and elevated lactate levels are associated with an oxygen debt or anaerobic metabolism, and can be rapidly assessed by a clinical laboratry or with portable point-or-care machines. Central venous blood with oxyhemoglobin saturation less than 70% (normal) indicates falling oxygen delivery relative to demand. Any centrally inserted lines including those inserted for dialysis or chronic care (PICC lines) can yield samples suitable for analysis. Acute elevations in the anion gap or lactate should be regarded as a sign of oxygen supply/demand imbalance until proven otherwise. 

Disruption in the economy of oxygen supply/demand imbalance is due to either excessive consumption, inadequate delivery or a combination of both (Table 2). In practice, while both VO_2_ and DO_2_ may be altered, most cases of oxygen debt result for problems with oxygen delivery, and the search for causes should begin there. As indicated in Figure 2, decreases in oxygen delivery result from either a decrease in cardiac output or in red cell mass.

### 2.4. Diagnosis of Hypovolemia and Hemorrhage

Low stroke volume states can be divided into two broad categories: (1) a low intravascular volume leading to low stroke volume and (2) a normal or high intravascular volume that cannot be converted to a normal stroke volume due to obstruction, inefficient ventricular filling, pump dysfunction, intracardiac abnormality, or valvular incompetence. The “low circulating volume” class of low stroke volume shown in Figure 2 would be revealed by absence of visible peripheral and neck veins, and in some cases monitor findings such as decreased pulse pressure, greater systolic blood pressure variability with mechanical ventilation, or low CVP [21, 22]. Orthostatic changes in blood pressure and heart rate have been used for decades to assess intravascular volume in patients able to undergo the maneuvers. McGee and colleagues reviewed the utility of the physical diagnosis in diagnosing hypovolemia in adults and found that pulse rate increases greater than 30 beats per minute and severe postural dizziness associated with severe (>1 L) hemorrhage was the only instance where postural maneuvers provided a reliable diagnoses [23]. The authors suggest that analysis of serum electrolytes and looking for an elevated BUN/creatinine is necessary for diagnosing other forms of hypovolemia and less severe hemorrhage. Improvement in blood pressure by movement to the supine position, Trendelenburg position, or after passive raising of the legs is indicative of a cardiac output that is responsive to fluids, and often adds additional support to suspicions of hypovolemia [24]. The standard approach to a patient with suspected hypovolemia is evaluation of the blood pressure response to rapid fluid challenge (a minimum of 0.5 L over 10 min). With the widespread use of portable transthoracic echocardiography, evaluation of inferior vena cava diameter and distensibility have emerged as early diagnostic tools, and have the advantage of avoiding fluid administration in cases where it may have no benefit [25, 26].

Significant blood loss as a cause of hypovolemia is diagnosed by sudden drops in hemoglobin, either alone or in the context of fluid administration. With instantaneous hemorrhage, isovolemic blood loss prior to fluid shifts would fail to reveal a depressed hematocrit, while still yielding the findings of increased low intravascular volume and increased vascular tone. Investigation would then lead to the etiologies of low cardiac output. With consideration and ruling out of a heart rate problem contributing to low CO, classes of stroke volume would then be considered. One may not yet arrive at the conclusion of hemorrhage, but would at least have the confidence that a low cardiac output due to a low preload is present, and begin infusion of fluids. A subsequent recheck of hematocrit during the fluid resuscitation would reveal a significant drop in this value, and promptly indicate hemorrhage. If blood loss was chronic or occurred more that 30 minutes prior to analysis, then interstitial to vascular fluid shifts would cause hemodilution and reveal a drop in hematocrit, and the cause of the elevated lactate and oxygen supply/demand imbalance would clearly be hemorrhage.

### 2.5. Evaluation of Other Low Cardiac Output States

Cardiac output is a factor of both oxygen delivery and blood pressure; thus an abnormally low blood pressure, or a VO_2_/DO_2_ imbalance may arise from ineffective cardiac output. In reasoning through the roots of hypotension, the patient would have either a low CO or low SVR. Extremity warmth, brisk capillary refill, and hyperdynamic circulation are findings associated with a low SVR. Conversely, cold extremities, weak pulses with narrow pulse pressures, and delayed capillary refill would provide an indication that vascular tone is abnormally high, and most likely as an attempt to compensate for a low cardiac output. Low CO would again lead to consideration of heart rate and stroke volume, and causes of low stroke volume. Enlarged neck veins are characteristic of all low stroke volume syndromes except frank hypovolemia, which was previously discussed. Other classes of low stroke volume states include the following.

1. Precardiac obstruction, as typified by tension pneumothorax and cardiac tamponade.
2. Valvular obstruction, for example, mitral stenosis exacerbated by tachydysrhythmia.
3. Acute heart failure as may occur with acute myocardial infarction, end-stage cardiomyopathy, acute volume overload, acute aortic insufficiency, and decompensation of pulmonary hypertension.
4. Postcardiac obstruction such as pulmonary embolism or aortic valve stenosis compounded by heart failure.
5. Mitral regurgitation.

Exam findings, monitor values and trends, and echocardiographic findings either alone or in combination would define which category of low SV is present. Findings from invasive monitors that differentiate the different syndromes are shown in red on Figure 2. Focused assessment by transthoracic echocardiography is becoming more and more common in intensive care units for purposes of evaluating these and other findings [27, 28].

### 2.6. Evaluation of Low Blood Pressure

In the case of low blood pressure with physical findings consistent with low vascular tone, a distributive shock picture can be assumed, and attention would then center upon further evaluation and empiric treatment of the conditions shown.

The utility of a physiologic evaluation as presented here is its ability to direct therapy appropriate to the class of abnormality once it is discovered. In distributive shock, for example, a patient with a low blood pressure clearly needs stabilization with a direct vasopressor. Because dilation of venous capacitance vessels accompanies profound arterial vasodilatation, fluids are also needed to restore preload. While the actual etiology of low vascular tone is not always apparent, the initial focus on the physiologic abnormality facilitates its rapid stabilization while diagnostic efforts continue. 

Likewise, if the evaluation leads to a low cardiac output state with compensatory vasoconstriction, the physiologic analysis should attempt to differentiate whether the low CO was due to hypovolemia or one of the other classes of abnormalities discussed above. Evidence of a low oxygen delivery in the setting of volume overload indicates a need for inotropic support while the underlying condition is investigated further. For all of these cases, identifying that the patient is maximally vasoconstricted should lead away from using vasopressors, which would not create any meaningful improvement in the patient's condition.

### 2.7. Making the Diagnosis

The tree branch diagram in Figure 2 in not only useful for understanding hemodynamic abnormalities in terms of perturbations of their subcomponents, but also demonstrates how specific diagnoses can emerge from such analysis. For example, a cold vasoconstricted patient with a metabolic acidosis and collapsed neck veins is suffering from frank hypovolemia or hemorrhage; careful observation of hemoglobin concentration during hydration will establish the diagnosis of hemorrhage. The other advantage of this physiologic approach is its consideration of all possibilities for a given category of abnormality. In our ICU for example, mitral regurgitation has been misdiagnosed as a pneumonia on a few occasions when abundant clinical evidence of a low cardiac output state was ignored in the presence of a chest X-ray appearing as a lobar pneumonia.

### 2.8. Endpoints of Resuscitation

Much controversy exists as to the most desirable endpoints for resuscitation from shock [29–33]. Rather than targeting specific numeric indices of oxygen delivery and cardiac output, a “bare minimal” goal should be to at least assure that an adequate blood pressure has been restored, and that oxygen delivery is not limiting consumption. While the curves in Figure 1 are impossible to generate at the bedside, they nonetheless provide a reasonable model for resuscitative goals. For example, one can see that a blood pressure that is disproportionately supported by a high vascular resistance will do so at the expense of cardiac output, which will have disastrous consequences for providing oxygen delivery to the tissues. Likewise, a complete focus on improving oxygen delivery with fluids and inotropes and without regarding the patient's blood pressure needs, may impair the function of end organs such as the kidneys [34]. Indeed, meeting these minimums is the basis of the hemodynamic resuscitation strategy advocated by the society of critical care medicine [35, 36]. Generally, an MAP of >65 mmHg is recommended; however, some patients autoregulate at a much higher range, and may require some individualization of this goal. Clearance of lactate and normalization of oxygen extraction ratios or central venous oxygen saturation are indications of an adequate oxygen supply/demand relationship. 

As resuscitation proceeds, it is important to continually reexamine VO_2_/DO_2_ and adequacy of blood pressure. Likewise, missed diagnoses (bleeding, myocardial infarction) and therapeutic mistakes (vasoconstrictors used instead of fluid) can be caught early in their evolution if these two physiologic relationships are frequently reevaluated. For example, a hypotensive patient with dehydration is given a vasopressor to elevate his blood pressure; a reevaluation of the serum studies showing a rising lactate and drop in pH may reveal that the initial perception of the problem was incorrect and that means of maintaining blood pressure with a greater contribution of CO over SVR should be pursued.

### 2.9. Teaching Physiology

On critical care rounds one commonly hears statements such as: “If Mr. X becomes hypotensive tonight, we will give a vasoconstrictor.” Statements such as the above are a good entry point for a discussion of whether the low blood pressure comes from a low cardiac output or loss of vascular tone, and how one can differentiate these possibilities and their etiologies. A lecture on shock given during the ICU rotation uses the principles presented here as framework for understanding shock states and their treatment. Laminated cards containing the dendrogram in Figure 2 are given to the students and residents as a cognitive aid to be used in the analysis of unstable patients.

## 3. Discussion

The methods used by different individuals to arrive at a diagnosis from a set of presenting conditions can be highly variable. Indeed, many texts have been written on diagnostic reasoning and the steps taken by master clinicians to solve challenging cases. Despite the breadth of work on the subject, there is no consensus on how clinical reasoning should proceed, or how students and young clinicians should be trained. There is a compelling body of evidence suggesting that in a growing number of conditions, delays and inefficiency in identifying critical problems and initiating treatment compromise survival [1, 2, 17, 37–39], and that outcomes of critical illness can be improved when treated in an organized manner with a greater sense of urgency [40–42]. 

This article presents an approach to the evaluation and initial treatment of critically ill patients that seems to satisfy a number of requirements posed by unstable patients at risk for organ failure.

1. It is comprehensive in the sense that it is based on well-known physiologic alterations that underlie all known classes of shock and organ dysfunction. As such, it includes the evaluation of VO_2_/DO_2_, which is often ignored by clinicians, to the detriment of their patients.
2. The method is efficient. Evaluation of vascular tone, blood pressure trends, hemoglobin content, acid-base status, and lactate production can be evaluated in a few minutes, while a patient's interview and exam occurs in parallel.
3. Diagnostic studies can be prioritized. If a stroke volume problem is suspected, one will know that obtaining an echocardiogram will be most useful in defining the etiology of the problem while physical exam can further narrow the set of possibilities. Understanding which studies contain the highest yield and building the use of diagnostic studies into a decision tree further improves efficiency and speed of care.
4. It does not delay treatment. Gross abnormalities in vascular tone, intravascular volume can be identified in less than a minute and acted upon promptly in an effort to stabilize the patient. Additional diagnostic impressions can be gained from the response to such early treatments. For example, poor responses to vasopressor infusions may suggest concomitant adrenal insufficiency while a brisk increase in blood pressure to fluid infusion may confirm the suspicion of blood loss.
5. It may improve diagnostic accuracy and efficiency. The key to defining deviations from normal physiologic values is that many diagnoses are coupled with specific patterns of abnormalities (see Figure 2). Arrival at a given pattern may not necessarily establish a specific diagnosis as quickly, but knowing the various possibilities to consider would hopefully prompt one to consider the advantages and disadvantages of treating multiple conditions while these possibilities are being evaluated. For example, identification of low blood pressure with low vascular resistance can be due to sepsis or a large inflammatory burst, adrenal crisis, or a severe allergic reaction. In some cases, it may be prudent to provide presumptive treatment for all three conditions, and not delaying therapy for want of a definitive diagnosis.
6. Finally, physiology is a fairly universal language. The care of the critically ill often involves discussions with many different disciplines including surgeons, nephrologists, cardiologists, general internists. Fragmentation of care, however, has the other unfortunate consequence of compromising patient safety and survival [43, 44]. Having a universal language to describe the approach to patient management may help with the development of an awareness of a patient's problems and management strategy amongst a diverse group of care providers.

The validity and efficacy of an educational strategy is difficult to directly evaluate especially with respect to changes in patient outcomes or other care process measure, and none has been attempted here. The tools and strategy presented have been formulated from careful consideration of the roots of critical illness, from instances where ICU admissions resulted from incomplete consideration of the relevant physiology, and from cases where slow diagnostic and therapeutic efforts have compromised patient survival [1, 2, 17, 37–39]. Aside from a subjective assessment of the efficacy of this approach, it is equally satisfying to demonstrate to trainees that most of the knowledge needed for successful evaluation of critically ill patients is already in their possession, and that with some “dusting off,” it can be used to understand and communicate about critical illness on a daily basis.

## Figures and Tables

**Table 1:** Typical hemodynamic changes associated with three accepted categories of shock. Arrows show degree of change from baseline in blood pressure (BP), cardiac output (CO), systemic vascular resistance (SVR), and cardiac preload. Additionally, alterations in the relationship between oxygen delivery and demand (DO_2_/VO_2_) and mean arterial pressure and organ blood flow (MAP/OBF) are indicated. The asterisk (*) indicates the primary abnormality associated with each shock state.

Type of shock	BP	CO	SVR	Preload	DO_2_/VO_2_	MAP/OBF
Cardiogenic	nl-*⇓*	*⇓⇓* ⇓*	*⇑⇑* *⇑*	nl-*⇑⇑*	*⇓⇓* *⇓*	nl-*⇓*
Hypovolemic	nl-*⇓*	*⇓*	*⇑⇑* *⇑*	*⇓⇓* ⇓*	*⇓⇓* *⇓*	nl, *⇓*
Distributive	*⇓⇓*	nl, *⇑⇑*	*⇓⇓* ⇓*	*⇓*	nl-*⇓*	*⇓⇓* *⇓*

Abbreviation: nl = normal range; arrows show increases or decreases.

**Table 2:** Disruptions in the normal economy of oxygen supply and demand can have profound impacts on solid organ function and long-term outcomes. Typically, changes in metabolic mode from aerobic to anaerobic as evidenced by high lactate or decreased central oxyhemoglobin saturation (sCVO_2_) can be explained by changes in either supply, demand, or both. Except in rare instances of hypermetabolism, most pathology in oxygen supply/demand can be traced to problems with delivery (DO_2_).

Increased demand (VO_2_)	Decreased supply, delivery (DO_2_)
*Uncommon*	Hemorrhage
Thyrotoxicosis	Decreased cardiac output
Malignant hyperthermia	Decrease heart rate
*Common*	Decreased stroke volume syndromes
Fever	Low blood volume
Systemic inflammation	Dehydration
Shivering, thermogenesis	Hemorrhage
Muscle contraction, fighting	Normal or elevated blood volume
	Heart failure, cardiogenic shock
	Inadequate filling time, tachycardia
	Valvular obstruction
	Valvular insufficiency
	Pulmonary hypertension
	Obstructive shock
	Pneumothorax
	Pulmonary embolus
	Cardiac tamponade

## Abbreviations

VO_2_: Oxygen uptake
DO_2_: Oxygen delivery
CO: Cardiac output
MAP: Mean arterial pressure
SVR: Systemic vascular resistance
CVP: Central venous pressure
PaOP: Pulmonary artery occlusion (wedge) pressure
mm Hg: Millimeters of mercury pressure.
